# Changes in treatment of intracranial aneurysms during the last decade in a large European neurovascular center

**DOI:** 10.1007/s00701-024-06064-4

**Published:** 2024-04-10

**Authors:** Francesco Calvanese, Anna Maria Auricchio, Anni Pohjola, Ahmad Hafez, Ville Nurminen, Miikka Korja, Jussi Numminen, Martin Lehecka, Rahul Raj, Mika Niemelä

**Affiliations:** 1 Department of Neurosurgery, University of Helsinki and Helsinki University Hospital, Haartmaninkatu 4, Po Box 320, 00029 HUS Helsinki, Finland; 2 Department of Neurosurgery, Fondazione Policlinico Universitario A. Gemelli IRCCS, Università Cattolica del Sacro Cuore, Rome, Italy

**Keywords:** Intracranial aneurysm, Subarachnoid hemorrhage, Surgical clipping, Endovascular treatment, Epidemiology

## Abstract

**Objective:**

Treatment modality for ruptured and unruptured intracranial aneurysms has shifted during the last two decades from microsurgical treatment towards endovascular treatment. We present how this transition happened in a large European neurovascular center.

**Methods:**

We conducted a retrospective observational study consecutive patients treated for an unruptured or ruptured intracranial aneurysm at Helsinki University Hospital during 2012–2022. We used Poisson regression analysis to report age-adjusted treatment trends by aneurysm location and rupture status.

**Results:**

A total of 2491 patients with intracranial aneurysms were treated (44% ruptured, 56% unruptured): 1421 (57%) surgically and 1070 (43%) endovascularly. A general trend towards fewer treated aneurysms was noted. The proportion of patients treated surgically decreased from 90% in 2012 to 20% in 2022. The age-adjusted decrease of surgical versus endovascular treatment was 6.9%/year for all aneurysms, 6.8% for ruptured aneurysms, and 6.8% for unruptured aneurysms. The decrease of surgical treatment was most evident in unruptured vertebrobasilar aneurysms (10.8%/year), unruptured communicating artery aneurysms (10.1%/year), ruptured communicating artery aneurysms (10.0%/year), and ruptured internal carotid aneurysms (9.0%/year). There was no change in treatment modality for middle cerebral artery aneurysms, of which 85% were still surgically treated in 2022. A trend towards an increasing size for treated ruptured aneurysms was found (*p* = 0.033).

**Conclusion:**

A significant shift of the treatment modality from surgical to endovascular treatment occurred for all aneurysm locations except for middle cerebral artery aneurysms. Whether this shift has affected long-term safety and patient outcomes should be assessed in the future.

**Supplementary information:**

The online version contains supplementary material available at 10.1007/s00701-024-06064-4.

## Introduction

The prevalence of unruptured intracranial unruptured aneurysms is reported to be as high as 6.6% in the general population [15]. However, only a minority of unruptured aneurysms will rupture and cause subarachnoid hemorrhage (SAH), the SAH incidence being approximately 6 per 100,000 [10]. Following SAH, 24% of the affected die outside of the hospital wards (e.g., at home, in ambulances, or in emergency rooms) and overall 39% die within 1 month of SAH [3]. With only a few natural history studies on unruptured aneurysms [14, 21, 49], decisions about prophylactic treatment are based on aneurysm-specific characteristics such as aneurysm location, size, morphology, and specific patient characteristics such as age, smoking status, and comorbidities [11].

Surgical treatment of intracranial aneurysms was first described almost 100 years ago [8] and remained the first-line choice for both the treatment of ruptured and unruptured intracranial aneurysms until 2002, when the ISAT trial reported more favorable functional outcomes following endovascular versus surgical treatment for ruptured intracranial aneurysms [26]. The trial results—that showed improved functional outcomes in good grade (World Federation of Neurosurgical Societies grade I–III) SAH after endovascular versus surgical treatment from an internal carotid (ICA) or posterior circulation aneurysm—were heavily extrapolated to include all intracranial aneurysms, independent of location, rupture status, or used endovascular technique [2]. While there is a paucity in the level of evidence supporting the shift from surgical to endovascular treatment, the shift has continued [37]. The transition towards endovascular treatment has happened at different paces depending on the local traditions and circumstances. Nowadays, endovascular treatment is the dominant treatment modality for intracranial aneurysms in developed countries. The same trend has happened in the invasive treatment of coronary disease where angioplasties and stents have replaced bypasses in the majority of patients [9, 50].

In this study, we analyzed the transition period from microsurgical to endovascular treatment in one high volume European neurovascular center. We aimed to explore how different aneurysm locations, aneurysm size, and rupture status affected the shift of treatment modalities.

## Methods

### Study setting and patient population

We conducted an observational retrospective study including all consecutive patients treated for unruptured or ruptured saccular intracranial aneurysms at Helsinki University Hospital (Helsinki, Finland) from January 2012 to December 2022. Helsinki University Hospital is the only neurosurgical unit covering a population of approximately 2.2 million people in Southern Finland (40% of the total population in Finland). It is the largest university hospital in the country and one of the largest hospitals in Europe with a well-known neurovascular practice.

We screened the electronical hospital records for all patients who were treated for either a ruptured or an unruptured intracranial aneurysm during the study period. We manually reviewed all cases and excluded the patients with aneurysms related to arteriovenous malformations, dural arteriovenous fistulas, or moyamoya disease. We only included patients whose aneurysm was treated.

The study was approved by the local research board and conducted according to the Strengthening the Reporting of Observational Studies in Epidemiology (STROBE) recommendations [47].

### Data collection

We extracted the data from electronic healthcare records and the picture archiving communication system. We recorded patient age at the time of treatment, sex, date, and modality of treatment (i.e., surgical versus endovascular), whether multiple aneurysms were treated in the same session, location of aneurysm(s), maximum size of the aneurysm(s), and aneurysm rupture status. We defined aneurysm maximum size as the largest diameter. For surgically treated patients, we measured the maximum diameter from the preoperative CT angiography because surgically treated patients rarely undergo digital subtraction angiography (DSA) in our institution. For endovascularly treated patients, we measured the maximum diameter from the projectional 2D DSA images and in case of a large partly thrombosed aneurysm, the maximum diameter was measured from pre-interventional CT angiography or MR images.

We classified endovascular treatment into coiling (including stent-assisted coiling and balloon-assisted coiling), intrasaccular device, flow diversion, and parent artery occlusion. We classified surgical treatment into clipping, proximal artery ligation, trapping, wrapping, or bypass. Due to the low number of patients undergoing proximal artery ligation, trapping, and wrapping, and their surgical similarities, these procedures were grouped for the analyses. We categorized intracranial aneurysm location into ICA, anterior communicating artery + A1 segment of the anterior cerebral artery (ACOM + A1), pericallosal artery, M1 segment of the middle cerebral artery (M1), middle cerebral artery (MCA) bifurcation or distal MCA (MCA-bif + distal MCA), vertebral artery + basilar artery + posterior cerebral artery (VBA + PCA), and posterior inferior cerebellar artery + anterior inferior cerebellar artery + superior cerebellar artery (PICA + AICA + SCA).

### Statistical methods

We presented categorical data as numbers with percentages, normally distributed data as means with standard deviations (SD) and non-parametric data as medians with interquartile range (IQR). Due to the large sample size and inherited group disparities, we did not test for significance levels between groups for baseline characteristics.

We calculated the annual change in treatment modality (surgery vs. endovascular) using marginal effects after fitting a Poisson regression model adjusting for patient age, checking for overdispersion. For the Poisson regression analyses, statistical significance was considered if the 95% confidence interval (CI) did not overlap 0.0. We used a Jonckheere-Terpstra test to test for trends in aneurysm size during the study period.

## Results

### Patient population

From January 2012 to December 2022, a total of 2491 patients with intracranial aneurysms were treated. Of these, 1421 patients (57%) were treated surgically and 1070 patients (43%) endovascularly. The median age was 58 years (IQR 49–65), 65% were women, maximum aneurysm size was 6 mm (IQR 4–8), and 44% were treated for a ruptured aneurysm (Table 1). Age and sex distributions were similar between the surgically and endovascularly treated patients. More patients in the endovascular group versus surgical group were treated for a ruptured aneurysm (53% vs. 37%). The most common aneurysm locations for surgically treated patients were MCA-bif or distal MCA (53%), ACOM + A1 (15%), and ICA (13%). The most common aneurysm locations for endovascularly treated patients were ACOM + A1 (36%), ICA (34%), and VBA + PCA (16%).

**Table 1:** Patient and aneurysm characteristics

Variable	All (*n* = 2491)	Surgery (*n* = 1421)	Endovascular (*n* = 1070)
Patient age, median (IQR)	58 (49, 65)	57 (49, 64)	58 (49, 66)
Sex			
Female	1627 (65%)	940 (66%)	687 (64%)
Male	864 (35%)	481 (34%)	383 (36%)
Aneurysm status			
Ruptured	1093 (44%)	523 (37%)	570 (53%)
Unruptured	1398 (56%)	898 (63%)	500 (47%)
Multiple aneurysms treated in same session	140 (6%)	105 (7%)	35 (3%)
Aneurysm location*			
ICA	552 (22%)	186 (13%)	366 (34%)
ACOM, A1	607 (24%)	218 (15%)	389 (36%)
M1	121 (5%)	108 (8%)	13 (1%)
MCA-bifurcation or distal MCA	787 (32%)	759 (53%)	28 (3%)
Pericallosal	102 (4%)	62 (4%)	40 (4%)
VBA, PCA	230 (9%)	61 (4%)	169 (16%)
PICA, AICA, SCA	92 4%)	27 (2%)	65 (6%)
Aneurysm maximum size*, median (IQR)	6 (4, 8)	7 (5, 9)	5 (4, 8)

*Abbreviations*: *ICA* internal carotid artery, *ACOM* anterior communicating artery, *M1* M1 segment of middle cerebral artery, *MCA* middle cerebral artery, *VBA* vertebrobasilar artery, *PCA* posterior cerebral artery, *PICA* posterior inferior cerebellar artery, *AICA* anterior inferior cerebellar artery, *SCA* superior cerebellar artery

^*^Largest aneurysm if multiple were treated in the same session

Clip ligation (97%) was by far the most common surgical treatment technique. Coiling (73%) followed by flow diversion (16%) and intrasaccular device (10%) were the most employed endovascular techniques. Patient and aneurysm characteristics for the different surgical and endovascular treatments are displayed in eTable 1 and 2.

### Overall unadjusted treatment trends

**Fig. 1:**
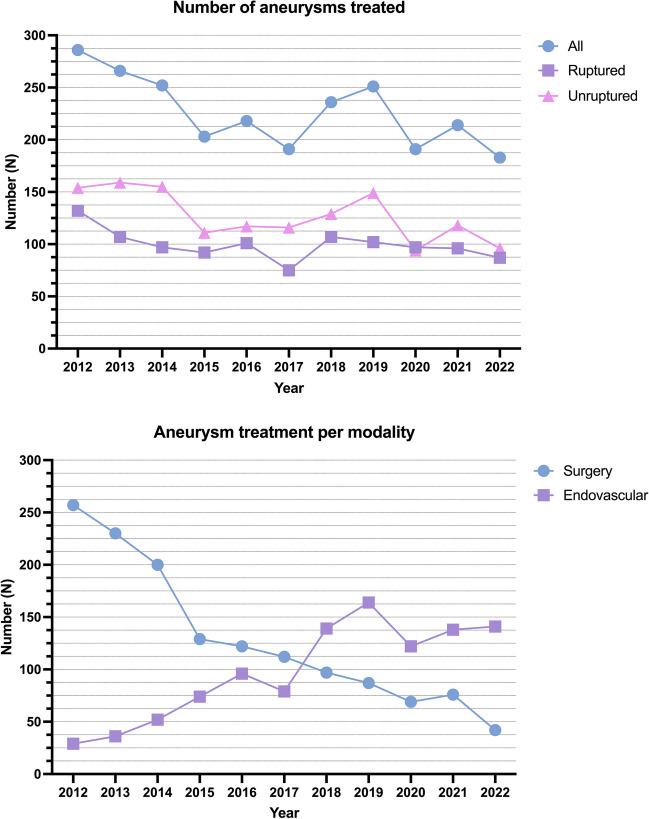
Absolute number of ruptured and unruptured aneurysms treated during the study period (upper) and absolute number of ruptured and unruptured aneurysms treated surgically or endovascularly (lower)

There was a general decreasing trend in the number of aneurysms treated from 2012 to 2022 (decrease with 7 aneurysms per year, 95% CI 4 to 10, Fig. 1). In 2012, only 10% of all aneurysms were treated endovascularly, after 2017 over 50%, and at the end of 2022 almost 80% of all aneurysms, respectively (eFigure 1). A rapid shift from surgical to endovascular treatment of posterior circulation and anterior circulating aneurysms (excluding all MCA aneurysms) occurred after 2014, whereas surgical treatment has remained the dominating modality for MCA aneurysms (eFigure 1). The median size for clipped aneurysms was somewhat larger than the median size for endovascularly treated aneurysms (eFigure 2). Regarding endovascular treatment modality, the use of flow diverters increased drastically for unruptured ICA aneurysms from 2018 onward (eFigure 3).

### Unadjusted treatment trends for ruptured and unruptured aneurysms by location

**Fig. 2:**
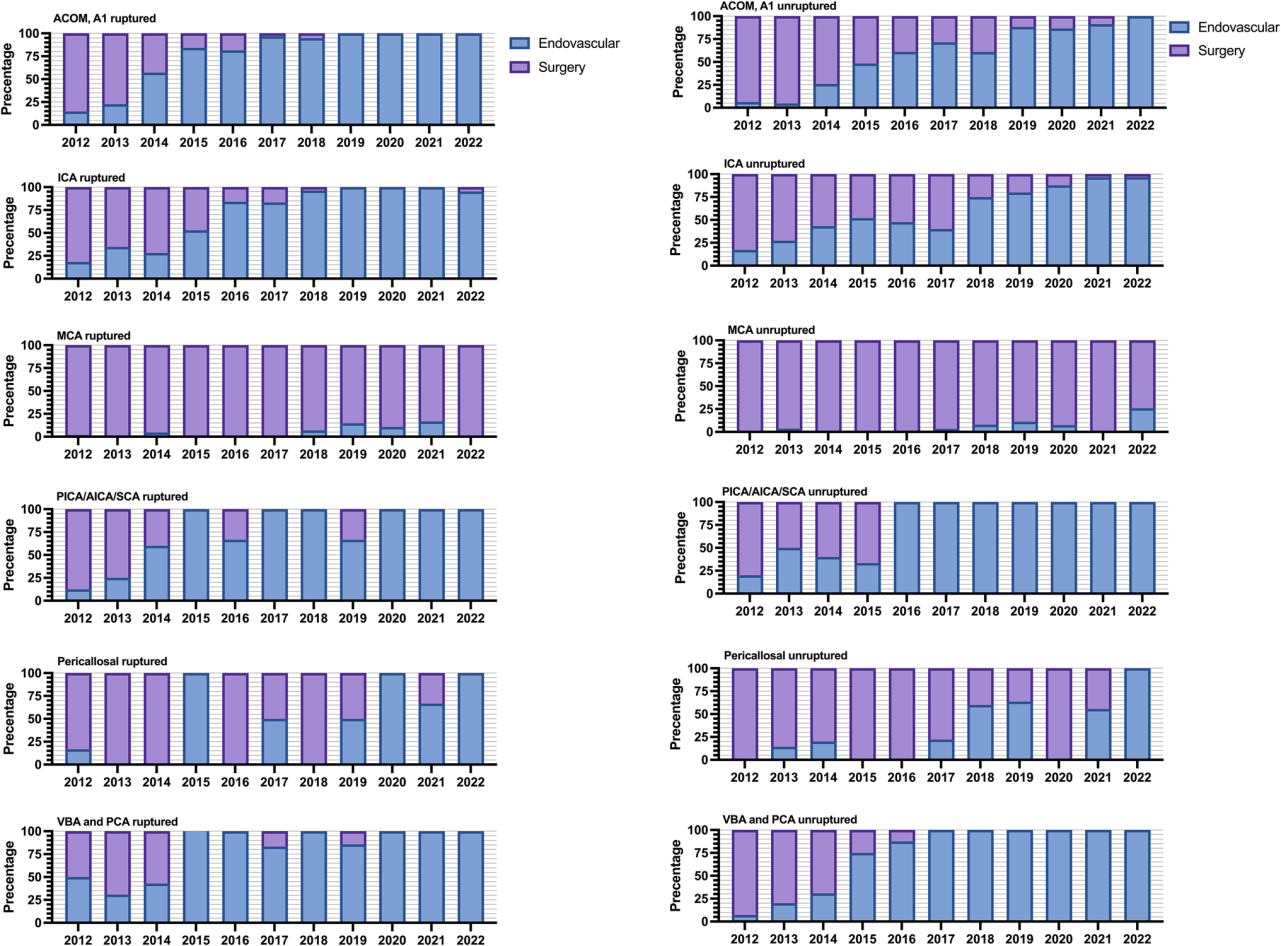
Proportion of aneurysms treated endovascularly and surgically per aneurysm location and rupture status

We observed a clear shift from surgical to endovascular treatment for all other locations except for MCA aneurysms occurred during the study period (Fig. 2). For all locations where the treatment modality changed, it happened first for the ruptured aneurysms followed later by the unruptured aneurysms. Although the general trend showed a decrease in the number of treated aneurysms, surgical treatment for ruptured MCA aneurysms and endovascular treatment for ruptured and unruptured ACOM + A1 and ICA aneurysms remained stable (eFigure 4). There was a trend towards larger sizes for ruptured but not unruptured aneurysms (*p* for trend = 0.003 and 0.078, respectively) (eFigure 5).

### Poisson regression

Age-adjusted trends confirmed the results of the unadjusted analysis. Regarding all aneurysms (ruptured and unruptured), the total annual rate of surgical treatment decreased by 6.9% compared to endovascular treatment. The decrease was most evident in unruptured VBA + PCA aneurysms (10.8%/year), unruptured ACOM + A1 aneurysms (10.1%/year), ruptured ACOM + A1 aneurysms (10.0%/year), and ruptured ICA aneurysms (9.0%/year). There was no change for MCA aneurysms (M1, MCA-bif + M2 distal). Due to the relatively low sample size, no statistically significant change was noted when separately analyzing ruptured and unruptured PICA + AICA + SCA (ruptured *n* = 62, unruptured *n* = 30) and pericallosal aneurysm (ruptured *n* = 33, unruptured *n* = 69), although the change was significant when combining unruptured and ruptured aneurysms in the same locations. Still, the confidence estimates suggest a strong trend towards a shift from surgical to endovascular treatment in these locations as well (Table 2).

**Table 2:** Poisson regression results showing the age-adjusted annual percentage point change of surgical compared to endovascular treatment for all ruptured and unruptured aneurysms

Aneurysm status	Ruptured and unruptured	Ruptured	Unruptured
Aneurysm location	Annual change in percentage points (95% CI)		
All locations	− 6.9 (− 8.4 to − 5.3)	− 6.8 (− 9.0 to − 4.5)	− 6.8 (− 9.0 to − 4.7)
ACOM and A1	− 10.1 (− 13.2 to − 7.0)	− 10.0 (− 14.0 to − 5.9)	− 10.1 (− 14.9 to − 5.3)
ICA	− 8.3 (− 11.4 to − 5.2)	− 9.0 (− 13.8 to − 4.2)	− 7.9 (− 12.0 to − 3.8)
M1	− 1.7 (− 9.3 to 5.9)	2.0 (− 13.9 to 17.8)	− 2.3 (− 11.3 to 6.6)
MCA-bif or M2 distal	− 1.0 (− 4.2 to 2.1)	− 1.3 (− 6.3 to 3.7)	− 0.9 (− 4.9 to 3.2)
PICA/AICA/SCA	− 7.8 (− 15.1 to − 0.6)	− 7.1 (− 15.7 to 1.5)	− 9.8 (− 24.0 to 4.3)
Pericallosal	− 7.8 (− 15.2 to − 0.4)	− 8.3 (− 20.6 to 4.1)	− 7.6 (− 17.0 to 1.7)
VBA and PCA	− 8.7 (− 13.4 to − 4.1)	− 7.0 (− 13.6 to − 0.3)	− 10.8 (− 17.4 to − 4.1)

Negative values refer to a decrease in surgical treatment compared to endovascular treatment. Positive values refer to an increase in surgical treatment compared to endovascular treatment

Statistical significance is achieved when the 95% confidence interval does not overlap 0.0

*Abbreviations*: *ACOM* anterior communicating artery, *A1* A1 segment of the anterior cerebral artery, *ICA* internal cerebral artery, *M1* M1 segment of the middle cerebral artery, *bif* bifurcation, *M2* M2 segment of the middle cerebral artery, *PICA* posterior inferior cerebellar artery, *AICA* anterior inferior cerebellar artery, *SCA* superior cerebellar artery, *VBA* vertebrobasilar artery, *PCA* posterior cerebral artery

## Discussion

In this large retrospective observational study, involving patients treated for either an unruptured or a ruptured intracranial aneurysm in a large European neurovascular center, we showed that the treatment of intracranial aneurysms shifted rapidly from surgical to endovascular care for all locations, except for the MCA aneurysms. The shift occurred earlier for ruptured than for unruptured aneurysms. The proportion of surgically treated patients decreased from 90 to 20%, with the decrease being most evident for posterior circulation aneurysms, ACOM aneurysms, and ICA aneurysms. At the end of the study period, endovascular treatment had essentially completely replaced surgical treatment for most aneurysms except for MCA aneurysms.

We noticed a general decrease in the number of treated intracranial aneurysms, both ruptured and unruptured. It has been speculated that the incidence of ruptured aneurysms has decreased as a result of successful prophylactic treatment of high-risk unruptured aneurysms [37]; however, it is more likely a consequence of decreased rates of smoking and hypertension [16, 22, 44, 45]. Moreover, the declining treatment rate of unruptured aneurysms is also affected by local policies; for example, treatment is nowadays rarely offered to patients with small aneurysms in locations that rarely rupture (e.g., paraopthalmic aneurysms [19]) or to elderly patients whose treatment-related complication rate surpasses the risks related to the natural history of the aneurysm [21]. Still, given the decreasing number of both the surgical treatment of intracranial aneurysms and the overall incidence, the challenge to maintain a competent surgical neurovascular center increases. Consequently, the European Stroke Organization have recommended the treatment of patients with unruptured intracranial aneurysms to be centralized to centers consulting more than 100 patients per year [11]. Whether this recommendation sufficiently assures high-quality aneurysm treatment, especially surgical treatment, remains unknown.

Our findings align with those of previous studies showing an increase in endovascular treatment over surgical treatment [2, 17, 23, 30, 37, 38]. Notably, the shift in our center occurred almost a decade later than in the USA (2007 vs. 2017) [2, 37]. The main reasons for the later shift can be attributed to the internal strong surgical neurovascular tradition and the need for evidence regarding the safety of endovascular treatment extending beyond a few years of treatment [18, 27, 39]. Still, when the shift took places, it was rapid, happening only over a few years’ time after the change of the chairman of the department in 2015. The swift change of treatment, especially for unruptured ACOM aneurysms, prompted us to oversee treatment results and complications. As could be anticipated, an increase in treatment-related complications of ACOM aneurysms could be seen in the beginning of the treatment shift, after which it decreased notably [12]. However, ACOM is only one of the several locations and similar studies looking at other locations are necessary to back up the shift from endovascular to surgical treatment. In an attempt to monitor the, probably unavoidable, shift towards endovascular treatment also for MCA aneurysms, we have launched a prospective quality of care study, assessing the safety of surgical and endovascular treatment for unruptured intracranial aneurysms (clinicaltrials.gov: NCT06147102).

At the end of our study period, approximately 90% of MCA aneurysms were still clipped. In comparison, approximately 55% of all MCA aneurysms are clipped in the USA [25]. Regionwide data from Europe is not available, but one study from Italy showed that 83% of MCA aneurysms were clipped [40]. One of the main rationales for maintaining surgical expertise for aneurysm clipping is possibility to simultaneously evacuate space-occupying intracerebral hemorrhages (ICH) in case of rupture. Especially for the MCA aneurysms, rupture is associated with a high risk of space-occupying ICHs requiring evacuation [1, 7, 29, 48]. Some authors have argued an endovascular treatment first and hematoma evacuation later approach, which in retrospective have yielded similar results to simultaneous surgical clipping and ICH evacuation [32, 36, 43]. Still, there are some inherited challenges to treat MCA aneurysms endovascularly, as they often are wide-necked and located close to vessel branches (M2 branches or M1 perforants) with poor collateral flow [33, 34]. Consequently, treatment-associated risks are higher and aneurysm occlusion rates are often lower than for other aneurysm locations treated endovascularly [24, 46] or compared to microsurgical clipping [35]. Still, there are studies showing good angiographic results and relatively low risks of complications following endovascular treatment for specific MCA aneurysms [4, 5, 13, 28, 31, 41, 51]. Given the current patient volumes of surgically treated aneurysm patients, even in high-volume neurovascular centers, and the challenges it poses to the training of future neurovascular surgeons, as well as the swift development of endovascular devices, it seems likely that a similar shift for MCA aneurysms will take place.

The use of flow diversion has dramatically changed the treatment of unruptured intracranial aneurysms, especially ICA aneurysms [6, 25]. For example, at the end of our study, over half of all unruptured ICA aneurysms were treated using a flow diverter stent, which is notably more than the 7% in the USA [25]. Of all flow diverters used for unruptured aneurysms in our study, 64% were for the treatment of ICA aneurysms, 15% for ACOM aneurysms, and 13% for VBA aneurysms. Given the continued technology improvement in flow diversion, their usage is likely to continue to increase, which also stresses the need for long-term (> 10 years) follow-up studies to evaluate the long-term effectiveness and safety.

In line with previous studies, our results verify the finding that the majority (57%) of ruptured aneurysms are small (< 7 mm) [19, 42]. However, in contrast to two previous studies conducted in Finland that showed a trend towards decreasing sizes for ruptured aneurysms from 1989–1995 to 2008–2009, we found a trend towards increasing sizes for ruptured aneurysms [19, 20]. The size of the unruptured aneurysms as well as the age of patients treated for an unruptured aneurysm remained stable throughout the study period, indicating that no major changes in the policy of treating unruptured aneurysms changed during the study period (eFigure 6).

### Limitations and strengths

We acknowledge some limitations of our study. The retrospective and single-center nature of the study limits its generalizability to other settings. Still, the healthcare system in Finland is publicly funded where all citizens have equal right to healthcare independent of insurance status and the physicians are without from personal incentives to treat patients, increasing the external validity of the study. The long study period (11 years) and manual validation of the data adds strength to the internal validity of the study. Be it noted that our study should not be confounded with a comprehensive epidemiological intracranial aneurysm or SAH study as we did not have data on patients diagnosed with unruptured intracranial aneurysms and who do not undergo treatment, nor on ruptured aneurysms not treated or people dying outside of the hospital. Further, our aim was to report trends in treatment modalities rather than outcomes. Thus, it remains unknown whether the noted shift from surgical to endovascular treatment has affected patient outcome.

## Conclusion

A significant shift of treatment from surgical to endovascular treatment occurred for all aneurysm locations except for middle cerebral artery aneurysms. With the decreasing overall incidence of intracranial aneurysms, low-volume centers might expect increasing difficulties to maintain microsurgical expertise of aneurysm treatment. Given the rapid change and relatively low level of long-term evidence the shift is based on, studies assessing long-term safety and patient outcomes should be conducted in the near future.

## Supplementary information

Below is the link to the electronic supplementary material.

Supplementary file1 eTable 1: Aneurysm and patient characteristics for surgically treated patients according to surgical technique. (DOCX 15 kb)

Supplementary file2 eTable 2: Aneurysm and patient characteristics for surgically treated patients according to endovascular technique. (DOCX 16 kb)

Supplementary file3 eFigure 1: Percentage of aneurysms treated surgically versus endovascularly (upper). Percentage of posterior circulation, middle cerebral artery (MCA) and anterior circulation (not MCA) treated surgically (middle) and endovascularly (lower). (PNG 31.8 mb)

High resolution image (TIF 3.75 mb)

Supplementary file4 eFigure 2: Median size of all aneurysms treated surgically (left) and endovascularly (right). (PNG 15.0 mb)

High resolution image (TIF 1.57 mb)

Supplementary file5 eFigure 3: Endovascular treatment technique for unruptured internal carotid artery aneurysms. (PNG 9.16 mb)

High resolution image (TIF 538 kb)

Supplementary file6 eFigure 4: Absolute number of aneurysms treated surgically (left column) and endovascularly (right column) according to aneurysm location. (PNG 56.9 mb)

High resolution image (TIF 3.50 mb)

Supplementary file7 eFigure 5: Median size of all aneurysms treated surgically and endovascularly (left), of all ruptured aneurysms treated surgically and endovascularly (middle) and of all unruptured aneurysms treated surgically and endovascularly (right). (PNG 22.6 mb)

High resolution image (TIF 2.71 mb)

Supplementary file8 eFigure 6: Patient age at the time of treatment of a ruptured aneurysm (left) or unruptured aneurysm (right). (PNG 15.1 mb)

High resolution image (TIF 318 kb)

## Data Availability

Due to legislative data transfer restrictions imposed by the European Union, the used dataset is not openly available for public use. However, qualified investigators can request access to the data from the Finnish Health and Social Data Permit Authority (findata.fi/en/).

## Code availability

Available upon request from the corresponding author.
