# Supplementary material for: Changes in treatment of intracranial aneurysms during the last decade in a large European neurovascular center
Source: Acta Neurochir (Wien). 2024 Apr 10;166(1):173. doi: 10.1007/s00701-024-06064-4 (PMC11004042; doi:10.1007/s00701-024-06064-4)
Supplement: Supplementary file 1 — Aneurysm and patient characteristics for surgically treated patients according to surgical technique. (DOCX 15 kb) [file 701_2024_6064_MOESM1_ESM.docx]

**eTable 1**: Aneurysm and patient characteristics for surgically treated patients according to surgical technique.

| **Variable** | **Clip ligation** (n=1376) | **Proximal artery ligation, trapping or wrapping** (n=11) | **Bypass and occlusion** (n=3) |
| --- | --- | --- | --- |
| **Patient age**, median (IQR) | 57 (49, 64) | 61 (55, 66) | 54 (27, 64) |
| **Sex** |  |  |  |
| Female | 924 (67%) | 4 (37%) | 12 (37%) |
| Male | 452 (33%) | 7 (64%) | 21 (64%) |
| **Aneurysm status** |  |  |  |
| Ruptured | 513 (37%) | 5 (45%) | 5 (15%) |
| Unruptured | 863 (63%) | 6 (55%) | 28 (85%) |
| **Multiple aneurysms treated in same session** | 105 (8%) | 0 | 0 |
| **Aneurysm location*** |  |  |  |
| ICA | 178 (13%) | 0 | 8 (24%) |
| ACOM, A1 | 215 (16%) | 0 | 3 (9%) |
| M1 | 105 (8%) | 0 | 3 (9%) |
| MCA-bifurcation or distal MCA | 743 (54%) | 3 (27%) | 13 (39%) |
| Pericallosal | 60 (4%) | 0 | 1 (3%) |
| VBA, PCA | 49 (4%) | 7 (64%) | 5 (15%) |
| PICA, AICA, SCA | 26 (2%) | 1 (9%) | 0 |
| **Aneurysm maximum size***, median (IQR) | 6 (4, 9) | 9 (5, 12) | 23 (15, 33) |
| *Largest aneurysm if multiple were treated in the same session  Abbreviations: ICA=Internal Carotid Artery, ACOM=Anterior Communicating Artery, M1=M1 segment of Middle Cerebral Artery, MCA=Middle Cerebral Artery, VBA=Vertebrobasilar artery, PCA=Posterior Cerebral Artery, PICA=Posterior Inferior Cerebellar Artery, AICA=Anterior Inferior Cerebellar Artery, SCA=Superior Cerebellar Artery | | | |
