# Supplementary material for: Changes in treatment of intracranial aneurysms during the last decade in a large European neurovascular center
Source: Acta Neurochir (Wien). 2024 Apr 10;166(1):173. doi: 10.1007/s00701-024-06064-4 (PMC11004042; doi:10.1007/s00701-024-06064-4)
Supplement: Supplementary file 2 — Aneurysm and patient characteristics for surgically treated patients according to endovascular technique. (DOCX 16 kb) [file 701_2024_6064_MOESM2_ESM.docx]

**eTable 2**: Aneurysm and patient characteristics for surgically treated patients according to endovascular technique.

| **Variable** | **Coiling**† (n=776) | **Intrasaccular device** (n=106) | **Flow diversion** (n=171) | **Parent artery occlusion** (n=17) |
| --- | --- | --- | --- | --- |
| **Patient age**, median (IQR) | 58 (50, 67) | 59 (52, 66) | 54 (44, 64) | 43 (35, 65) |
| **Sex** |  |  |  |  |
| Female | 503 (65%) | 64 (60%) | 113 (66%) | 7 (41%) |
| Male | 273 (35%) | 42 (40%) | 58 (34%) | 10 (59%) |
| **Aneurysm status** |  |  |  |  |
| Ruptured | 482 (62%) | 46 (43%) | 31 (18%) | 11 (65%) |
| Unruptured | 294 (38%) | 60 (57%) | 140 (82%) | 6 (35%) |
| **Multiple aneurysms treated in same session** | 32 (4%) | 0 | 3 (2%) | 0 |
| **Aneurysm location*** |  |  |  |  |
| ICA | 258 (33%) | 7 (7%) | 97 (57%) | 4 (24%) |
| ACOM, A1 | 309 (40%) | 56 (53%) | 23 (13%) | 1 (6%) |
| M1 | 9 (1%) | 1 (1%) | 3 (2%) | 0 |
| MCA-bifurcation or distal MCA | 16 (2%) | 5 (5%) | 4 (2%) | 3 (18%) |
| Pericallosal | 32 (4%) | 4 (4%) | 4 (2%) | 0 |
| VBA, PCA | 102 (13%) | 27 (25%) | 34 (20%) | 6 (35%) |
| PICA, AICA, SCA | 50 (6%) | 6 (6%) | 6 (4%) | 3 (18%) |
| **Aneurysm maximum size***, median (IQR) | 5 (4, 7) | 6 (4, 8) | 6 (4, 9) | 8 (4, 14) |
| *Largest aneurysm if multiple were treated in the same session  †Includes stent-assisted coiling and balloon-assisted coiling  Abbreviations: ICA=Internal Carotid Artery, ACOM=Anterior Communicating Artery, M1=M1 segment of Middle Cerebral Artery, MCA=Middle Cerebral Artery, VBA=Vertebrobasilar artery, PCA=Posterior Cerebral Artery, PICA=Posterior Inferior Cerebellar Artery, AICA=Anterior Inferior Cerebellar Artery, SCA=Superior Cerebellar Artery | | | | |
