# Supplementary figures and images for: Changes in treatment of intracranial aneurysms during the last decade in a large European neurovascular center
Source: Acta Neurochir (Wien). 2024 Apr 10;166(1):173. doi: 10.1007/s00701-024-06064-4 (PMC11004042; doi:10.1007/s00701-024-06064-4)

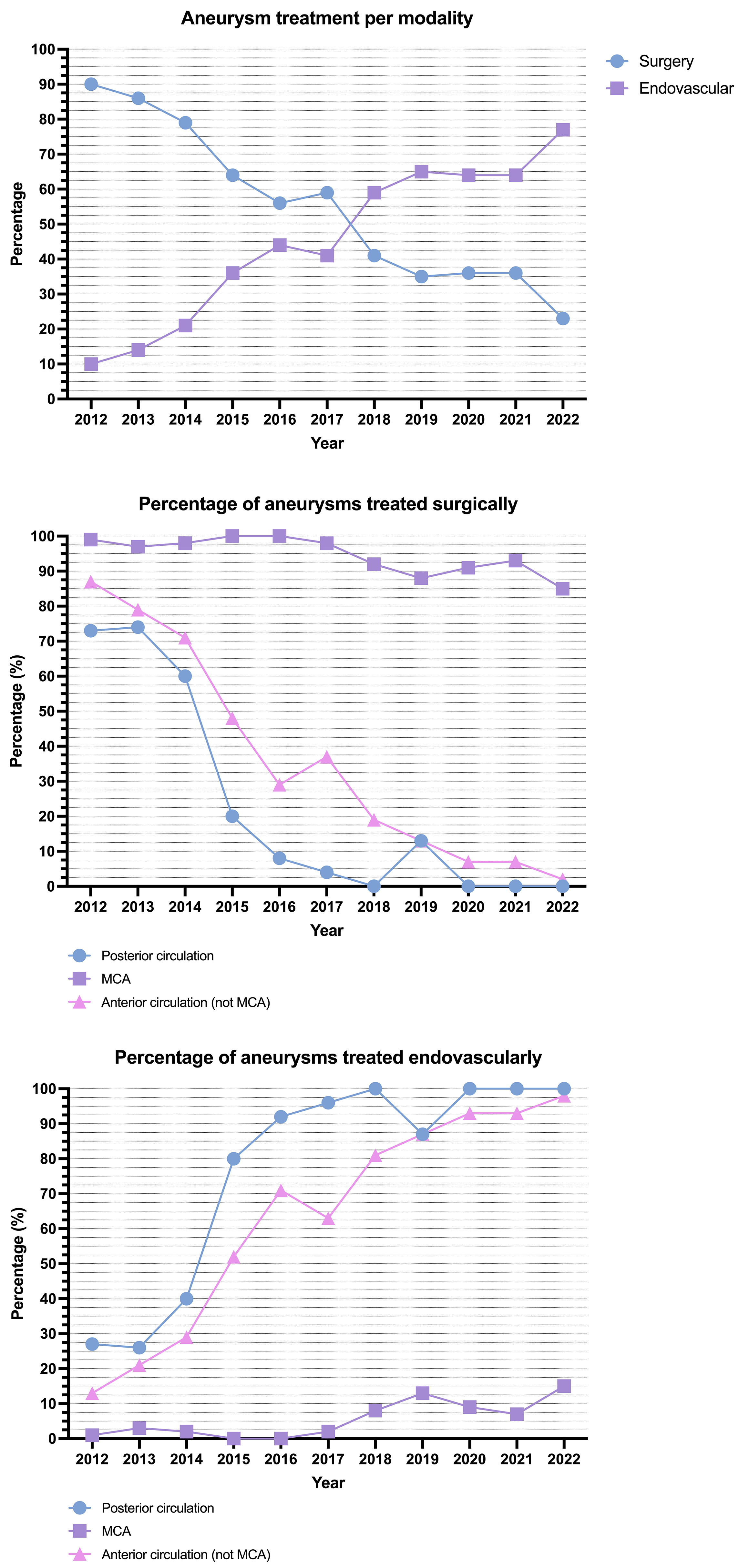

Supplement: Supplementary file 3 — Percentage of aneurysms treated surgically versus endovascularly (upper). Percentage of posterior circulation, middle cerebral artery (MCA) and anterior circulation (not MCA) treated surgically (middle) and endovascularly (lower). (PNG 31.8 mb) [file 701_2024_6064_Fig3_ESM.png]

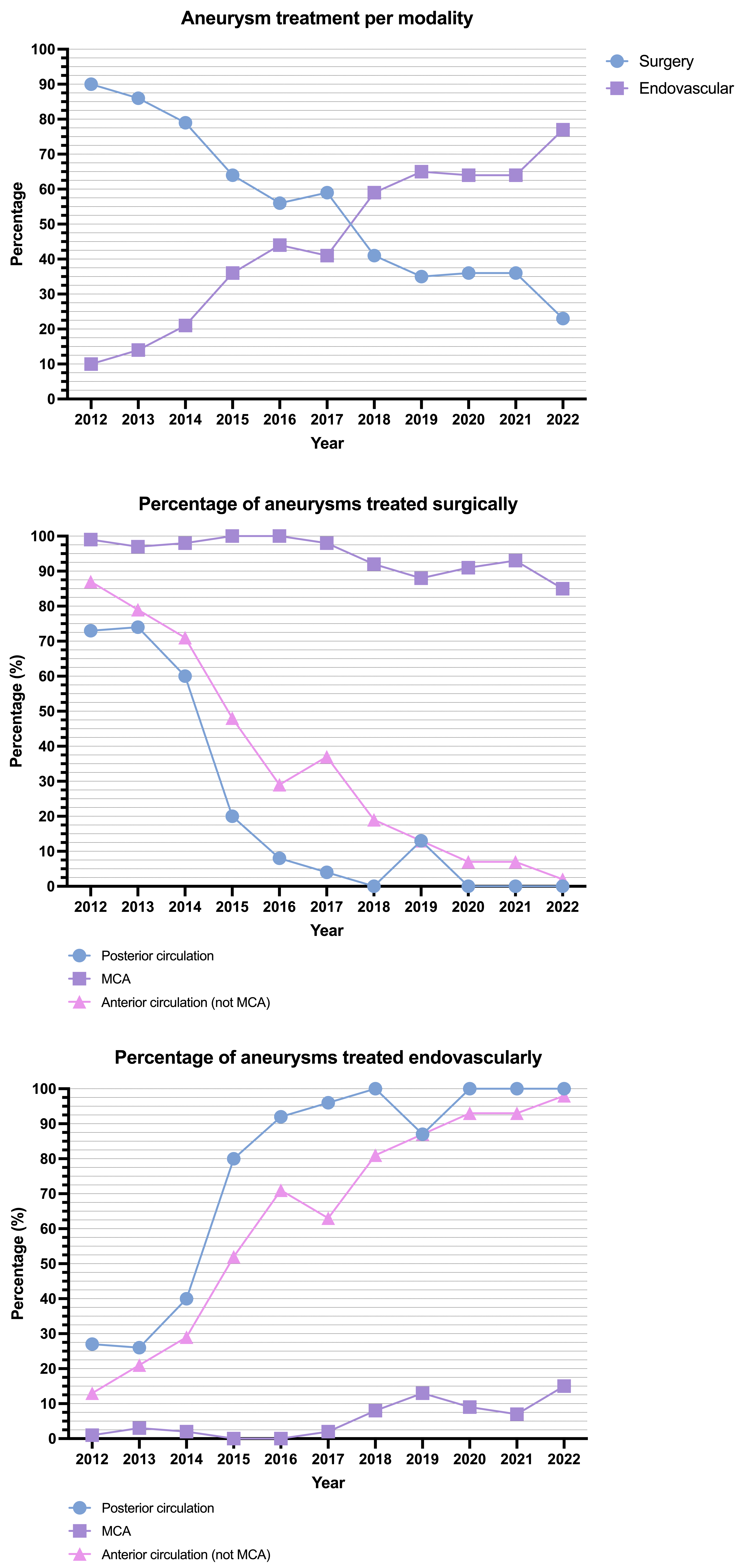

Supplement: Supplementary file 4 — High resolution image (TIF 3.75 mb) [file 701_2024_6064_MOESM3_ESM.tiff]

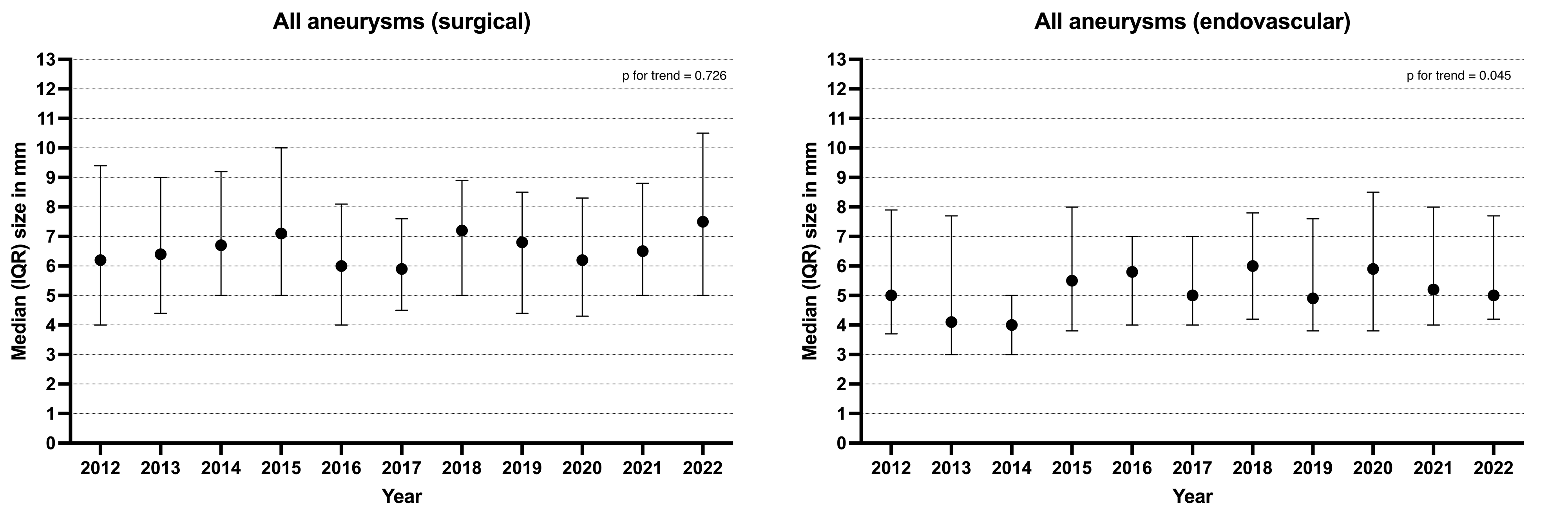

Supplement: Supplementary file 5 — Median size of all aneurysms treated surgically (left) and endovascularly (right). (PNG 15.0 mb) [file 701_2024_6064_Fig4_ESM.png]

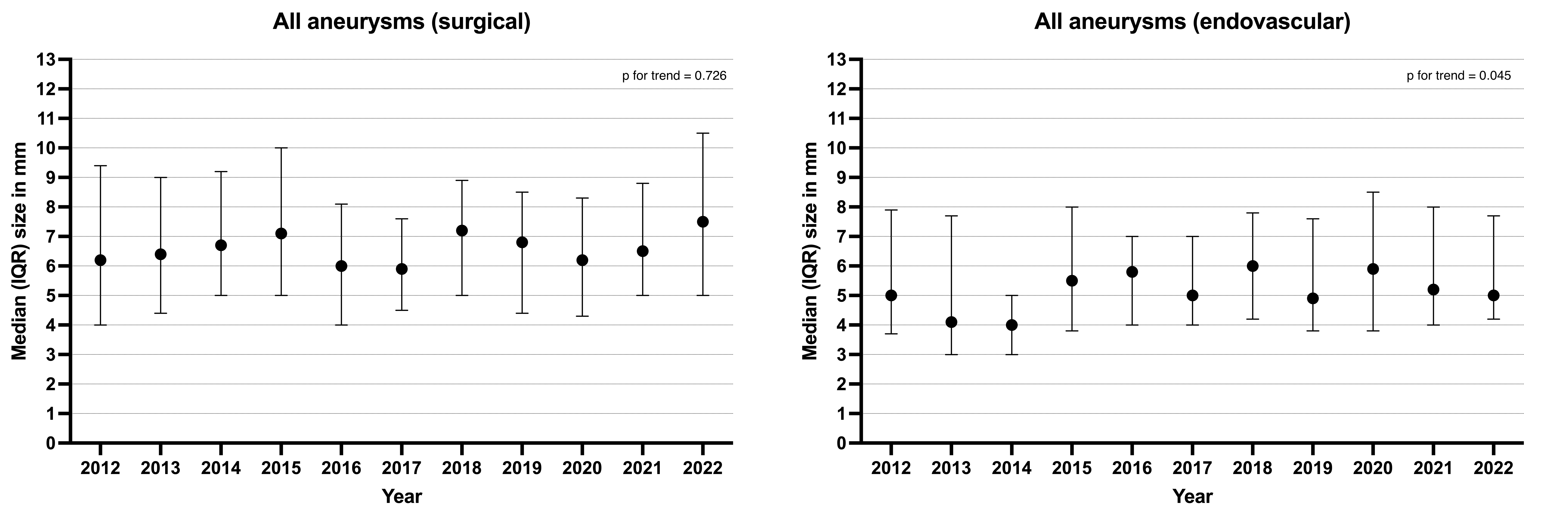

Supplement: Supplementary file 6 — High resolution image (TIF 1.57 mb) [file 701_2024_6064_MOESM4_ESM.tiff]

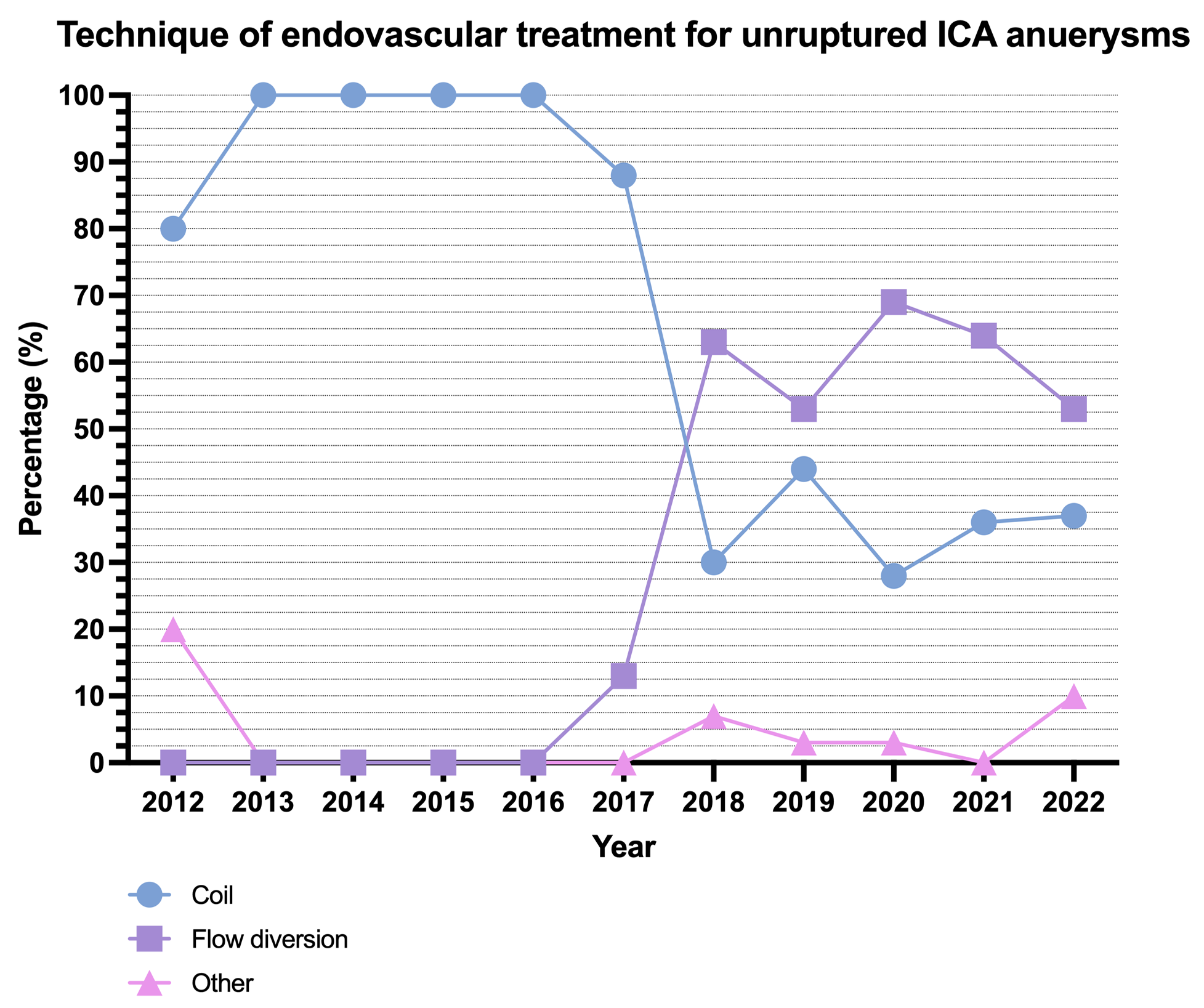

Supplement: Supplementary file 7 — Endovascular treatment technique for unruptured internal carotid artery aneurysms. (PNG 9.16 mb) [file 701_2024_6064_Fig5_ESM.png]

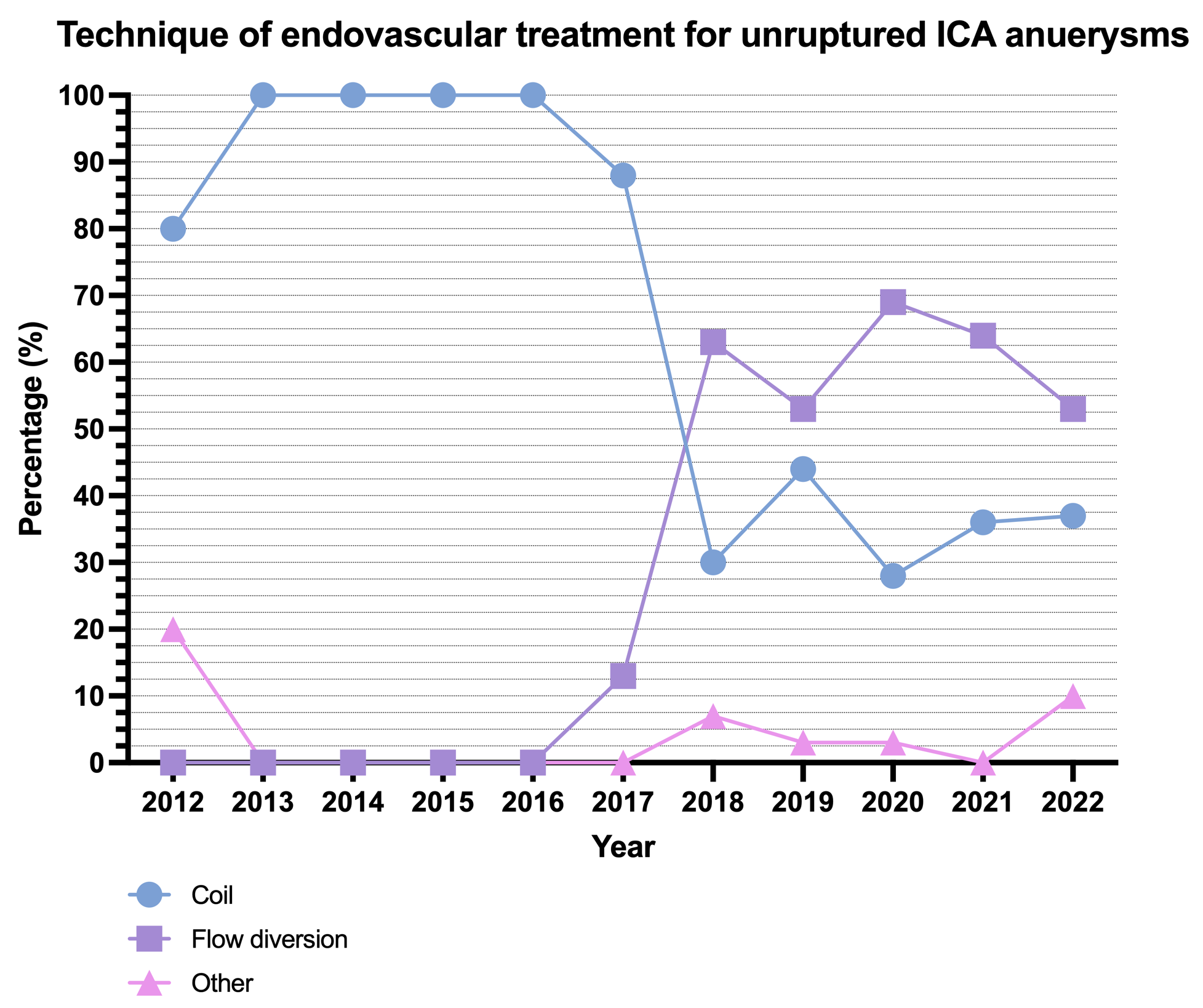

Supplement: Supplementary file 8 — High resolution image (TIF 538 kb) [file 701_2024_6064_MOESM5_ESM.tiff]

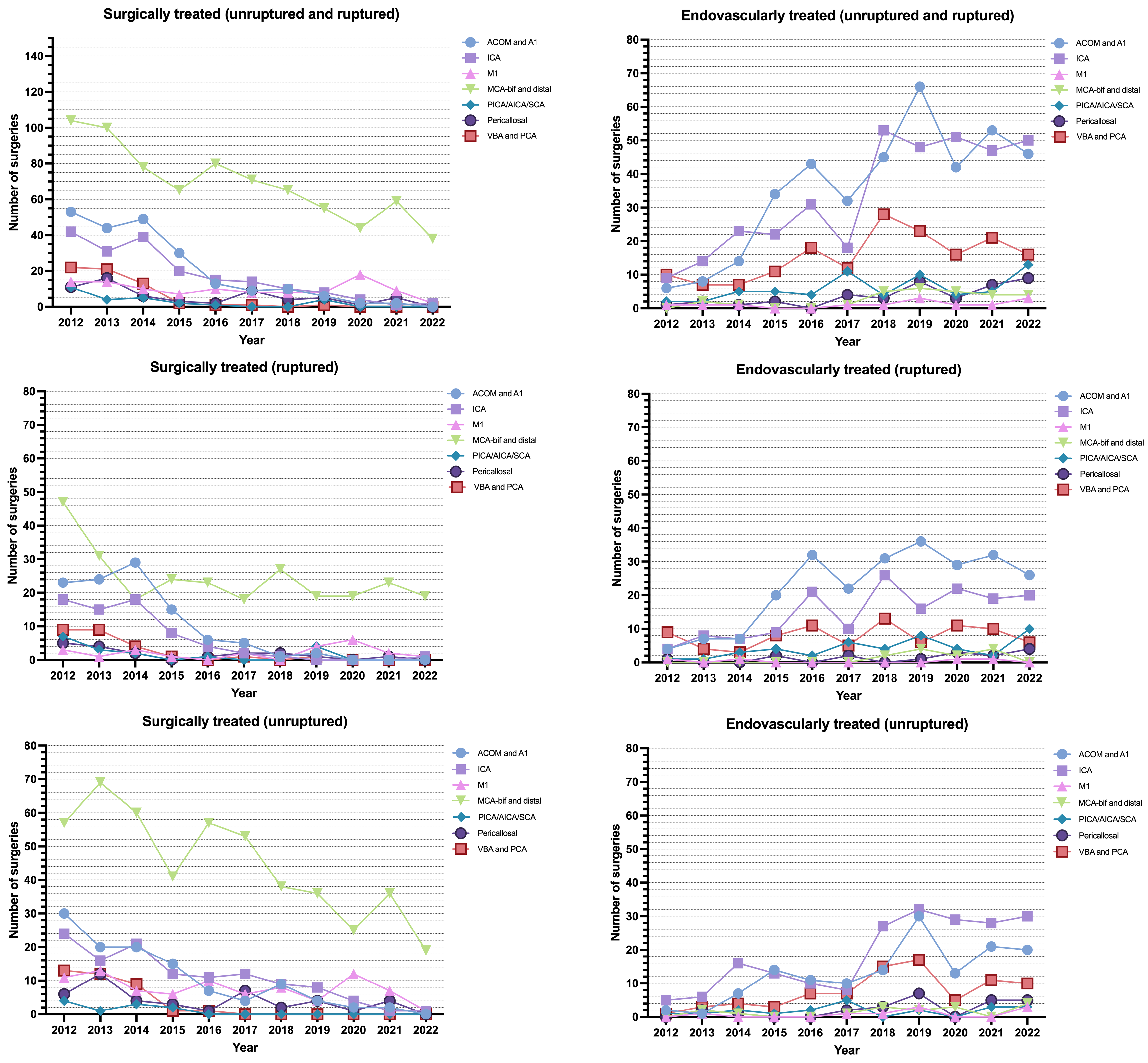

Supplement: Supplementary file 9 — Absolute number of aneurysms treated surgically (left column) and endovascularly (right column) according to aneurysm location. (PNG 56.9 mb) [file 701_2024_6064_Fig6_ESM.png]

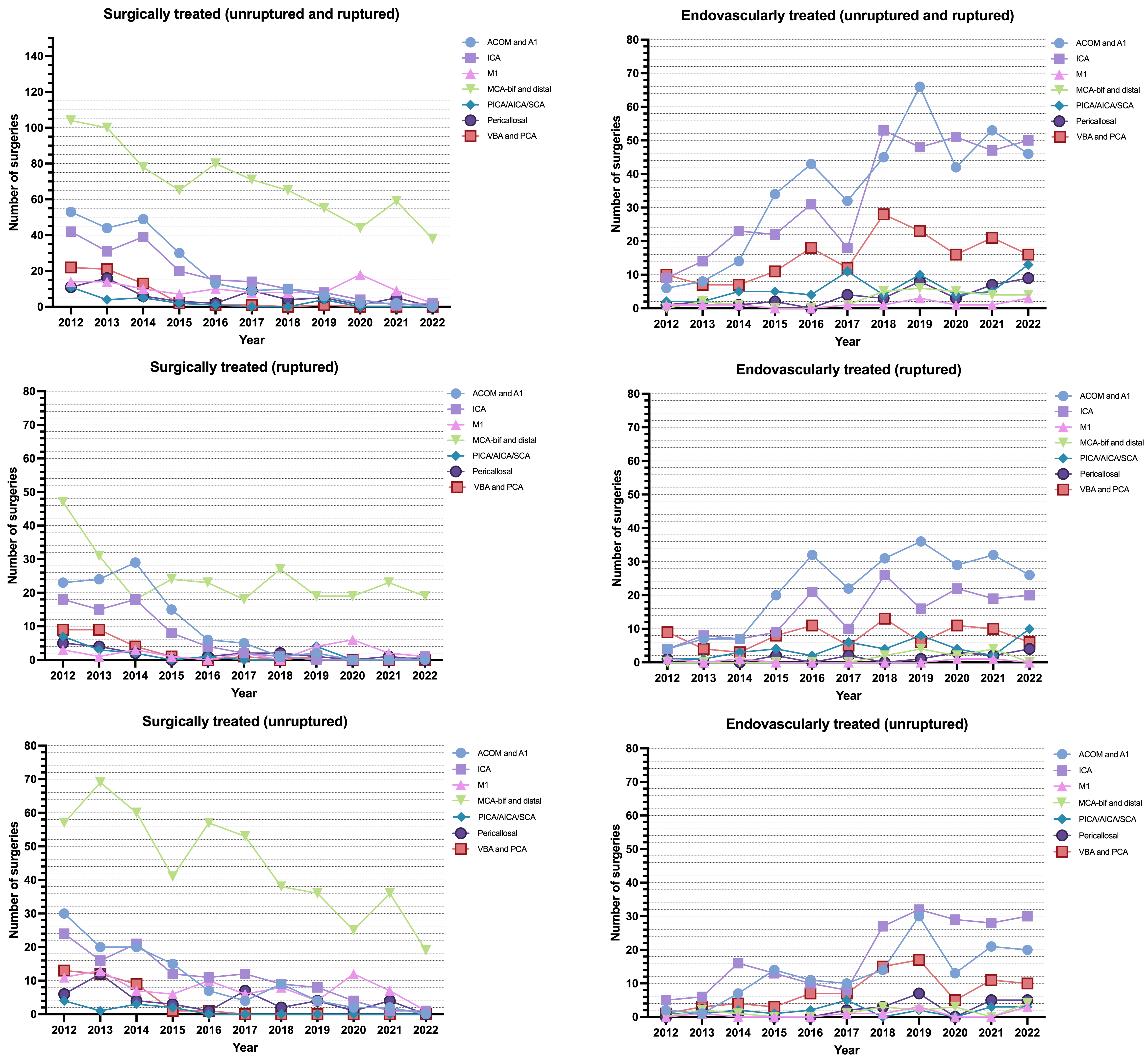

Supplement: Supplementary file 10 — High resolution image (TIF 3.50 mb) [file 701_2024_6064_MOESM6_ESM.tiff]

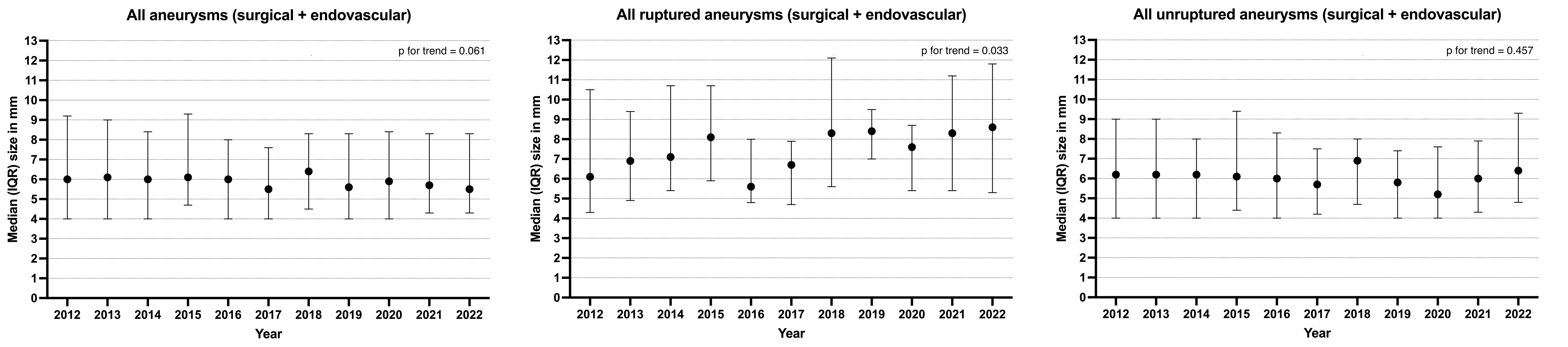

Supplement: Supplementary file 11 — Median size of all aneurysms treated surgically and endovascularly (left), of all ruptured aneurysms treated surgically and endovascularly (middle) and of all unruptured aneurysms treated surgically and endovascularly (right). (PNG 22.6 mb) [file 701_2024_6064_Fig7_ESM.png]

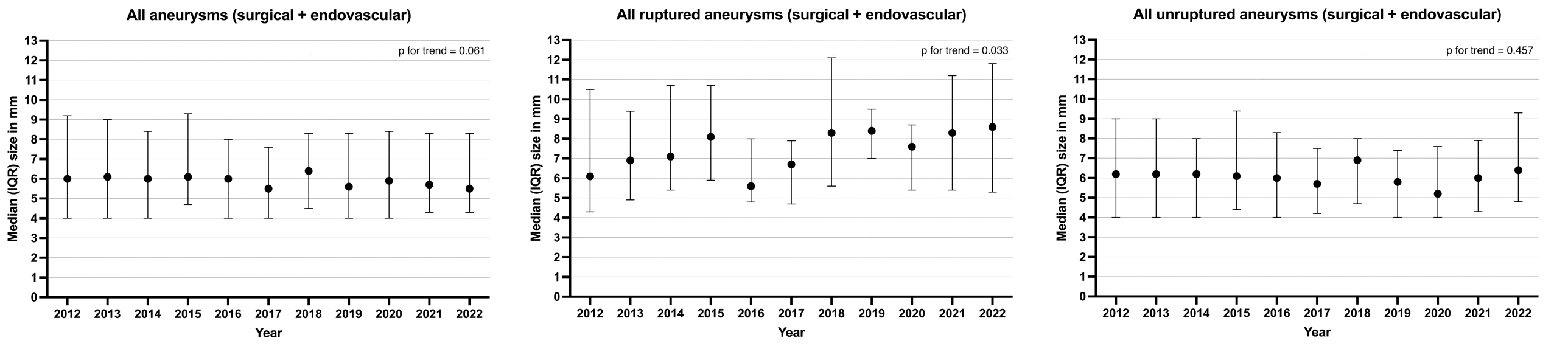

Supplement: Supplementary file 12 — High resolution image (TIF 2.71 mb) [file 701_2024_6064_MOESM7_ESM.tiff]

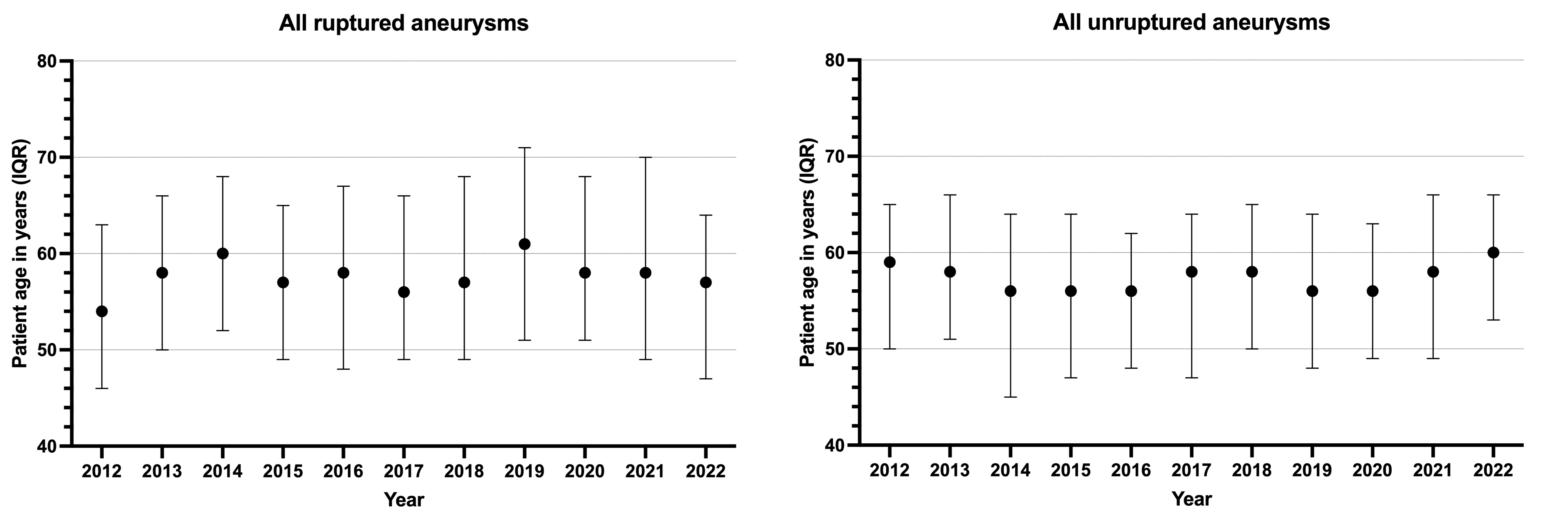

Supplement: Supplementary file 13 — Patient age at the time of treatment of a ruptured aneurysm (left) or unruptured aneurysm (right). (PNG 15.1 mb) [file 701_2024_6064_Fig8_ESM.png]

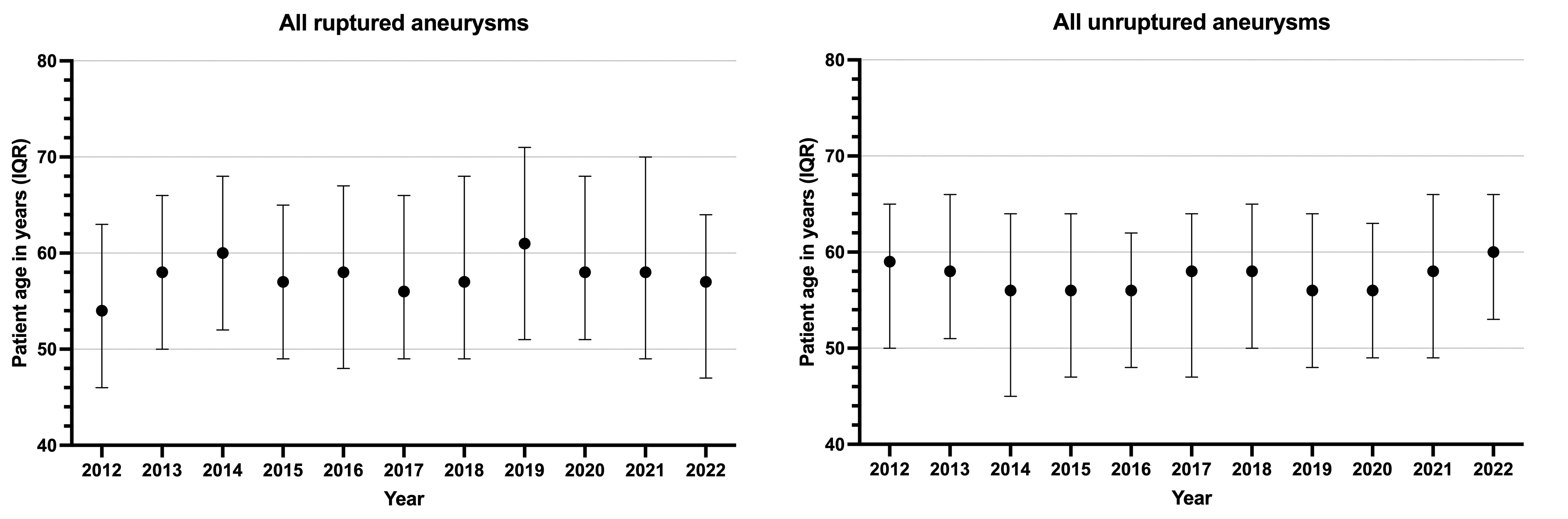

Supplement: Supplementary file 14 — High resolution image (TIF 318 kb) [file 701_2024_6064_MOESM8_ESM.tif]
